# How are population-based funding formulae for healthcare composed? A comparative analysis of seven models

**DOI:** 10.1186/1472-6963-13-470

**Published:** 2013-11-08

**Authors:** Erin Penno, Robin Gauld, Rick Audas

**Affiliations:** 1 Centre for Health Systems, Department of Preventive and Social Medicine, University of Otago, P.O. Box 913, Dunedin 9054, New Zealand; 2 Division of Community Health and Humanities, Faculty of Medicine, Memorial University of Newfoundland, St. John’s, NL A1B 3X9, Canada

**Keywords:** Health funding, Population based funding models, Comparative analysis, Resource allocation

## Abstract

**Background:**

Population-based funding formulae act as an important means of promoting equitable health funding structures. To evaluate how policy makers in different jurisdictions construct health funding formulae and build an understanding of contextual influences underpinning formula construction we carried out a comparative analysis of key components of funding formulae across seven high-income and predominantly publically financed health systems: New Zealand, England, Scotland, the Netherlands, the state of New South Wales in Australia, the Canadian province of Ontario, and the city of Stockholm, Sweden.

**Methods:**

Core components from each formula were summarised and key similarities and differences evaluated from a compositional perspective. We categorised approaches to constructing funding formulae under three main themes: identifying factors which predict differential need amongst populations; adjusting for cost factors outside of needs factors; and engaging in normative correction of allocations for ‘unmet’ need.

**Results:**

We found significant congruence in the factors used to guide need and cost adjustments. However, there is considerable variation in interpretation and implementation of these factors.

**Conclusion:**

Despite broadly similar frameworks, there are distinct differences in the composition of the formulae across the seven health systems. Ultimately, the development of funding formulae is a dynamic process, subject to availability of data reflecting health needs, the influence of wider socio-political objectives and health system determinants.

## Background

A drive towards more cost effective models of healthcare funding has seen many countries reassess traditional methods of provider payment, such as fee for service, in an effort to reorient incentives away from provider-induced demand. Prospective models of funding have emerged as an important means of incentivising efficiency, compelling healthcare providers to operate within set budgetary limits. In tandem, equity objectives have demanded greater recognition of the myriad of factors that drive health expenditure which are disproportionately distributed throughout populations. In response, many health policy makers have sought to develop population-based (or needs-based) funding formulas that seek to balance resource allocation with the relative costs of providing care to defined population groups.

Descriptions of the methodology used in developing funding formulae have been discussed at length elsewhere [1,2]. The literature is either dense in description and analysis of country-specific formulae and methods for how these might be calculated [3,4]. Where cross-country comparisons are made, these are at a very general level with countries considered on a case-by-case basis and limited comparative analysis [1,5], focused on a particular part of the world such as the Europe [6], or produced as government reports [7]. Moreover, much of the literature on funding formulae is now dated, having been published up to a decade ago, and there is a dearth of studies that consider the simple question of what is included in funding formulae, given availability of various options that policy makers may choose from. This is a substantial shortcoming in the literature. Funding formulae are increasingly seen as key methods for resource allocation, both within developing and developed country health systems [2,8]. Yet, few comparative descriptions of the structure of funding formulae exist. Additionally, there is limited information regarding the different components used in funding formulae and basic health system determinants.

For these reasons, and to examine the nuances of how policy makers in different jurisdictions construct funding formulae, we undertook a comparative analysis of the elements comprised in funding formulae in seven health systems, both extending on the work of others [1,5,6], as well as building an understanding of the contextual influences underpinning formulae construction. Using a compositional perspective, we explore key similarities and differences across seven funding formulae and contrast these against common demographic and geographic features. In this regard, this article is linked to a growing body of comparative research which seeks to gain insight into the reasons behind such differences and, in doing so, promote cross-national dissemination and learning [9-11].

## Methods

In this article we explore key similarities and differences across funding formulae in New Zealand, England, Scotland, the Netherlands, the state of New South Wales (NSW) in Australia, the Canadian province of Ontario, and the city of Stockholm, Sweden using the approach taken in 2012. The cases represent a convenience sample and were selected following a review of high-income health systems and jurisdictions that are predominantly publicly-financed and use a population-based funding formula. The key criterion for their selection was availability of published information or grey literature on the formulae. However, in order to evaluate responses to different health, geographic and demographic challenges we also sought to include a more diverse range of health system typologies and geographical regions than covered in prior studies [6]. In doing so, our sample is consistent with those found among the comparative health policy research literature [12-15].

The focus of our analysis is on acute and inpatient care components as these form the core of the formulae reviewed. In this respect we examine only those factors which are included in the final models of the funding formula, rather than factors which were considered but rejected by the different jurisdictions. The discussion highlights points of comparison and difference across a series of common formula characteristics, including how age, ethnicity, socioeconomic status and factors such as geography and otherwise unmet health care needs within the community are accounted for.

### The seven cases: background information

Health expenditure in each of the seven jurisdictions is predominantly publicly financed through general taxation, with the exception of the Netherlands where a mandatory private insurance scheme forms the basis of health care funding. In keeping with global concerns over cost containment, health plans^a^ in each jurisdiction are funded using global, prospective budgets and, in New Zealand, England, Scotland, NSW and Stockholm, are responsible for populations within defined geographic areas.

As shown in Table 1, a diverse range of demographic challenges confront the seven health systems. All have aging populations, with median age particularly high in the Netherlands, England and Scotland [16,17]. Population size varies from 51 million in England to just 1.9 million in Stockholm, with populations in all territories concentrated in urban areas [16,18-21]. However, population density across the entire region serviced by each health system varies significantly, ranging from only nine people per square kilometre in NSW to greater than 390 in England and the Netherlands [18,22]. Similarly, there are distinct differences in the degree of ethnic homogeneity within populations [16,23-25]. Income inequality is highest in New Zealand and England, with Sweden providing a divergent comparison, ranking well below the OECD average for income inequality [18].

**Table 1:** Seven healthcare systems background information

	**Health system**	**Pop (mil)**	**Pop density (per km2)**	** Urbanisation%**	**Median age**	**Main ethnic groups**	**Income inequality index (GINI coefficient)**^**(b)**^
**England**	Universal access funded by General Taxation	51.3 [18]	395 [22]	80^(a)^[16]	40^(a)^[16]	White 92.1%^(a)^[16]	0.34^(a)^[18]
						Black 2% Indian or	
						Pakistani 3.1%	
**Scotland**	Universal access funded by General Taxation	5.1 [18]	66 [18]			Mixed 1.2%	
						Other 1.6%	
**New South Wales**	Universal access funded by federal and state taxation mixed with optional private insurance coverage	7.1 [18]	9 [18]	89 [19]	37 [19]	White 92% [16,25]	0.30 [18]
						Asian 7%	
						Aboriginal 2.2%	
**Netherlands**	Mandatory social insurance	16 [18]	395 [18]	83 [16]	41 [16]	Dutch 80.7% [16]	0.27 [18]
	purchased from profit making insurers					EU 5%	
						Indonesian 2.4% Turkish 2.2% Surinamese 2% Moroccan 2% Caribbean 0.8% Other 4.8%	
**New Zealand**	Universal access funded by General Taxation	4.3 [18]	16 [18]	86 [16]	37 [16]	European 77% Maori 15% [23]	0.34 [18]
						Pacific 7%	
						Asian 10%	
**Ontario**	Universal access funded by General Taxation	12.9 [18]	14 [18]	85 [20]	39 [16]	77% Non minority [20]	0.32 [18]
						4% Black	
						14% Asian	
						2% Aboriginal	
**Stockholm**	Universal access funded by general and local taxation	1.9 [18]	299 [18]	95 [21]	39 [17]	9.6% Population foreign citizens, 39% classified as foreign born [17]	0.23 [18]
						Foreign-born or first-generation immigrants: Finns, Yugoslavs, Danes, Norwegians, Greeks, Turks	

Data Sourced From: ^(a)^*United Kingdom estimates*, ^(b)^*Country estimates*.

[16] (Central Intelligence Agency. 2011).

[17] (Statistics Sweden. 2011).

[18] (Organisation for Economic Co-operation and Development 2011).

[19] (Australian Bureau of Statistics. 2010).

[20] (Statistics Canada. 2009).

[21] (Statistics Sweden. 2010).

[22] (Office for National Statistics 2010).

[23] (New Zealand Ministry of Social Development 2010).

[24] (Statistics Canada. 2007).

[25] (Aboriginal Affairs NSW. 2007).

Pressure to maintain equitable funding which is responsive to the demand placed on different health plans has triggered each health system to develop population-based funding models. Population-based models were implemented in the 1970s in England and Scotland and 1980s in New Zealand and NSW, with current formulas representing the latest stage in an ongoing evolution [26-30]. Ontario’s nascent Health Based Allocation Model provides an interesting contrast, representing an innovative, but as yet unproven, model shaped by the increasing availability of individual clinical data.

## Results

### A comparison of the seven formulae

The seven jurisdictions demonstrate considerable differences in the structure of their funding formulae. Examining the seven from a compositional perspective, three recurring themes emerge. Firstly, all seven attempt to identify factors that are positively correlated with demand and, frequently, corresponding expenditure. By implication these factors are deemed to reflect health need. Secondly, most formulae seek to compensate health plans for costs that exist outside the scope of measures of health need alone. Thirdly, to align with equity objectives, some formulae incorporate an allowance for unmet need. The following sections explore the range of strategies included in funding formula in respect to each of these three key components, identifying common structural frameworks and contrasting the distinct approaches adopted in each of the seven jurisdictions.

### Need

Each formula starts with the idea of aligning allocations with factors that explain differences in demand; by implication these factors reflect differential health need. Tables 2 and 3 provide an overview of the components used to capture need in each of the seven formulae. In practice these factors fall under two broad categories: demographic indicators which act as proxies (Table 2) and more manifest measures of need, such as disease status (Table 3).

**Table 2:** Demographic measures included in the seven funding formulae

	***Age***	***Sex***	***Socioeconomic***	***Ethnicity***	***Geography***
**England **[29]	5 year age bands		Standardised no qualifications		Supply Index:^(c)^
	0-85+ years		Young people not staying in education		Disability living allowance claimants over 60
			Pension credit claimants		Mean Waiting Time
			Low birth weight at births		Access to admitted care providers
			Income Deprivation affecting children		Distance to outpatient providers
			Disability living allowance claimants		Distance to admitted patient providers
			Incapacity Benefit/Severe Disability Allowance Claimants		Number of GPs
					Accessibility for acute provider capacity
					Accessibility score for outpatient capacity
**Scotland **[31]	5 year age bands	Male	*Dependant on Care Programme*:		Maternity: Urban–rural classification (10 categories)
	0-90 + years	Female			
			Mental Health:% of people in one person households		
			% in social rented housing		
			Maternity: Mean house price		
**New South Wales **[26]	5 year age bands	Male	Index of Occupation & Education	% Aboriginal & Torres Strait Islanders	Accessibility/Remoteness index of Australia
	0-85 + years	Female			
	
**Netherlands **[32,33]	20 age categories	Male	Source of Income (Benefits, self employed, employed with insurance, no source of income)		Urbanisation
	0, 1-4, 5-9, 10-14…90+	Female			
			Average Income		Proximity of care facilities (per 1000 inhabitants within 25 km radius)
			Proportion of non-western immigrants		
			Marital Status		
**New Zealand **[34]	5 year age bands	Male	Deprivation quintiles based on Index of Deprivation (NZDep2006)	Maori, Pacific, Other	
	0-85 + years	Female			
**Ontario **[35]	4 age categories for person profile	Male	Quintile of Neighbourhood Income per Person Equivalent		Rurality (Non Rural, Rural, Very Rural)
		Female	(Dichotomised into Low SES (Quintile 1) and Higher SES (Quintiles 2-5))		Small Hospital (Yes/No)
	1-17, 18-59, 60-79, 80+				
	2 age categories for ranking clinical group				
	0-17, 18+				
			
**Stockholm **[36,37]	5 & 10 year age bands		Marital Status (Married or cohabitating with children, lone)		
	0-80+ years		House tenure (small houses, other)		
			Educational Level (At least upper secondary, lower education, missing data)		
			Employment Status (early retirement, other)		

^(c)^England’s Supply Index is set at national average rates in the final funding models, neutralising its effect on allocation.

Data sourced from:

[29] (Department of Health. 2011).

[31](NHSScotland Resource Allocation Committee. 2007).

[26] (New South Wales Health. 2005).

[32] (Ministry of Health Welfare and Sport 2008).

[33] (Ministry of Health Welfare and Sport 2012).

[34] (Ministry of Health. 2004).

[35] (Health System Information Management and Investment Division. 2011).

[36] (Andersson et al. 2000).

[37] (Andersson et al. 2010).

**Table 3:** Disease status measures included in the seven funding formulae

	**Clinical**	**Epidemiological**
**England **[29]		Age-specific death rate
		Standardised long term limiting illness
**Scotland **[31]		Acute Care:
		All causes standardised mortality 0-74 years
		Limiting long term illness rate
**New South Wales **[26]		Standardised Mortality Ratio >70
**Netherlands **[32,33]	Pharmacy Cost Groups (20 categories)	Standardised death probability
	Diagnosis Cost Groups (13 categories)	
**New Zealand**		
**Ontario **[35]	HBAM Inpatient Grouper (583 Groups)	
	Refined Clinical Groups (324 categories)	
	Major Clinical Groups (21 Groups)	
**Stockholm**		

Data sourced from:

[29] (Department of Health. 2011).

[31](NHSScotland Resource Allocation Committee. 2007).

[26] (New South Wales Health. 2005).

[32] (Ministry of Health Welfare and Sport 2008).

[33] (Ministry of Health Welfare and Sport 2012).

[35] (Health System Information Management and Investment Division. 2011).

#### Demography

##### Age and sex

Rather than measuring health needs directly, demographic characteristics are frequently used as proxy measures, drawing on easily classifiable population features positively correlated with cost to infer the expected annual expenditure of individuals. Age, the most obvious criterion, has been adopted as a universal predictor of need due to a clear association with expenditure. However, the degree of refinement between age bands varies widely throughout each of the seven formulae, ranging from up to 20 age categories in Scotland and the Netherlands to at most four categories in Ontario. The majority of formulae also consider sex as a simple prognostic variable. Yet its inclusion in funding formulae is more discretionary. Both Stockholm and England exclude sex as a major cost driver in acute care service areas, as its impact on costs becomes marginal once allocations are aggregated to area level [36,38].

##### Socio-economic status

In addition to these two most basic characteristics, the seven formulae universally reflect the impact of socio-economic factors on expected expenditure. The range of indicators used to measure socio-economic status (SES) varies widely, though small area indices reflecting socioeconomic elements such as welfare dependency, education level, income and housing are common across the seven health systems. In Ontario this is restricted to a neighbourhood level measure of income. Scotland also uses a comparatively limited range of indicators which are specific to each care programme. Interestingly SES is not explicitly included in the Acute Care component of the Scottish formula although other care programmes include indicators which reflect housing circumstance. In contrast, England draws on a wide range of indicators designed to reflect relative deprivation between health plans but customises the factors to reflect those most explanatory of need at different ages by varying, both the indicators and the weightings applied to those indicators according to five year age group [29]. With policy perhaps linked to relatively greater income inequalities [18] socio-economic circumstance as a predictor of health need is, by contrast, more influential in New Zealand, where an aggregate measure of deprivation forms one of four independent predictors of need [34]. Stockholm also relies heavily on indicators which are aligned with SES, although in contrast to every other system socio-economic factors were derived from individual level data with each selected variable demonstratively correlated with expenditure on health services [36].

##### Ethnicity

Overlapping but distinct from socio-economic factors [39,40], the impact of ethnicity on health status is recognised as an independent predictor of need in only two of the formulae. The clustering of New Zealand’s population into relatively few ethnic groups [23], combined with significantly worse health outcomes in Maori and Pacific populations compared with the rest of the population [41], has led New Zealand to treat ethnicity as a fundamental predictor of health status [34]. Similarly poor health outcomes among the indigenous populations in Australia means ethnicity is also a central concern in the NSW Health Needs Index [26]. Although ethnicity has been explored as a potential predictor in some other jurisdictions [7], it is either absent from the other five formulae, or, as is the case in the Netherlands, integrated into measures of socio-economic status.

##### Geography

Geography can play an important role in influencing both an individual’s health status and their access to health services [42]. Funding formulae offer a means to balance geographic disparities, although the process is fraught with the difficulty of differentiating legitimate factors which reflect genuine variation in need from spurious, supplier induced discrepancies in expenditure [5]. Possibly because of this complexity only five of the formulas directly link health need to a geographic classification. The geographical profile emphasised in a formula tends to be correlated with population density. In general, jurisdictions with a high population density emphasise the influence of urbanisation on health need. For example, the Netherlands, the most densely populated of the seven jurisdictions [18], encompasses geography by making a risk adjustment based on residence in one of ten regional clusters and which primarily reflects relative urbanisation, although this is closely tied to other adjustments based on socio-economic status. Stockholm, also a densely populated region [18], takes a more limited approach, adjusting for urbanisation based on residence in either “central districts” or “other” areas.

In contrast, rural geography is identified as an independent indicator of need in health systems with the lowest population densities [18]. Ontario’s emphasis on individual costs sees personal health profiles weighted according to three categories of rurality, which reflect the relative accessibility of health services and whether patients have sought care in a rural facility [35]. A similar emphasis on accessibility is seen in NSW. Greater admissions in remote and rural areas are seen as linked to increased need, as well as to reduced access to primary and specialist care coupled with greater distance from facilities, resulting in the substitution of community-based for hospital-based care. Hence, notwithstanding criticism that its inclusion would serve to reinforce inefficient provision practices, NSW places considerable weight on geographical remoteness in the needs component of its formula [26]. In this way, NSW allows supplier-induced variations in practice to influence allocations. Although also a sparsely populated jurisdiction, New Zealand is a notable exception to the practice of linking health need to geographic status, choosing not to include rurality as a predictor of need in its formula.

Capturing measures specifically tied to both accessibility and availability of services, rather than categorising health plans according to urban–rural status, England presents an entirely different approach to integrating geography into a formula. Stemming from concern their omission would produce inaccurate estimates of the needs variables, supply factors thought to influence utilisation patterns, and consequently need, support England’s needs framework. However, these variables are set to national average rates in the final model, in effect “sterilising” or equalising the influence of supply on demand. In so doing England imposes a standardised reference point for supply with the underlying assumption that any differences in utilisation between areas, after controlling for supply, are the result of genuine differences in health need [30].

#### Disease status

Population characteristics provide a straightforward means of disaggregating populations on the basis of expected expenditure. However, dependency on demography as a proxy for need is limited to New Zealand and Stockholm. In the other five formulae, demographic predictors of need are considered in conjunction with measures of disease status (Table 3).

##### Clinical measures

Where individual level data exists there has been scope to integrate personalised clinical profiles into formula based allocations. This is the case in Ontario where individual diagnoses rather than demographic characteristics form the core of the formula. The use of an electronic record for each resident of the province has enabled Ontario to develop unique ‘person profiles’ based on diagnostic and procedural episodes of the preceding three years. Using this data individuals are assigned to clinical groups which are ranked by case mix severity and aggregated to one of 21 Major Clinical Groups [35]. Efforts to minimise adverse risk selection have also led the Netherlands to make extensive use of clinical predictors, grouping individuals into pharmaceutical and diagnostic cost groups to capture the costs associated with chronic illness at both primary and secondary care level [32].

##### Epidemiological measures

In contrast, in jurisdictions where data are predominantly available at an area level, epidemiological information has been used to weight allocations. The simplest adjustment of this type is made in the NSW formula where a measure of premature mortality is incorporated as a factor in the formula’s needs index. Recognition that mortality was unlikely to correspond with morbidity across different areas has led England to draw on broader measures of health status that reflect both premature mortality and the burden of chronic disease [7]. As for the demographic factors in the formula, mortality and morbidity variables are customised by varying both the indicators and the weightings applied to those indicators according to five year age group to derive the most explanatory set of factors for each age group across all services areas [29]. The Scottish needs index also features both morbidity and mortality factors. However, these are restricted to explaining costs across all age groups in Acute Services only [31].

### Other cost factors

In addition to the relative need of their enrolled populations, health plans are often subject to cost pressures outside of those recognised in the capitation component of a formula. Almost all of the seven formulas make some adjustment for excess costs incurred by health plans (Table 4). Conceptually, these can be broken down into adjustments that correct inaccuracies in a health plan’s share of capitation funding and adjustments that address the impact of various market factors on the cost of supply.

**Table 4:** Additional cost components included in the seven funding formulae

	**Cost of supply**	**Capitation funding corrections**
**England **[29]	Market Forces Factor	Growth Area Growth Adjustment^(d)^
**Scotland**[31]	Unavoidable Costs	
**New South Wales **[26]	Teaching and Research	Interstate Flow
	State-wide and Selected Specialty Services	
	Dispersion Costs Factor	
	Small hospitals factor	
	Public/Private Hospital Mix	
	Private Hospital Activity Substitution	
**Netherlands **[32,33]		Retrospective Adjustments
**New Zealand **[34]	Rural Adjuster	Overseas Adjuster
**Ontario **[35]	Unit Costs ( Facility expenses, Facility type, Weighted service activity, Teaching, Specialised services, Rural geography, Size of Facility)	Market Share
**Stockholm**		

^(d)^England’s Growth Area Growth Points adjustment was discontinued in the 2011/12 funding allocation

Data sourced from:

[29] (Department of Health. 2011).

[31] (NHSScotland Resource Allocation Committee. 2007).

[26] (New South Wales Health. 2005).

[32] (Ministry of Health Welfare and Sport 2008).

[33] (Ministry of Health Welfare and Sport 2012).

[34] (Ministry of Health. 2004).

[35] (Health System Information Management and Investment Division. 2011).

#### Addressing cost of supply

Influenced by innumerable factors, the costs of supplying health care can vary substantially between health plans. Concern that these differences may arise out of systematic pressures in different areas has led to health systems organised on a geographic basis to include various compensatory measures in funding formulae in an effort to equalise legitimate supply costs between health plans.

The most comprehensive adjustments are found in Ontario and NSW. The dual public-private nature of the NSW health system has driven the inclusion of adjustments for the relative resource intensity of different patient types. By adjusting allocations for the lower expected costs of private patients as well as the higher than expected costs of providing referral and teaching services the NSW formula attempts to ensure that health plans are neither penalised nor advantaged for different categories of service provision [26]. A purely public system, Ontario nevertheless mirrors NSW, weighting allocations according to proportion of specialisation, cost-weighted activity and teaching costs [35]. Over and above differential needs related to remote populations both formulae also include adjustments for the additional costs of providing facilities in remote areas [26,35].

In other sparsely populated jurisdictions [18] rurality is the central factor in determining cost of supply. In New Zealand, the emphasis has been on offsetting the additional costs that arise from providing services to remote areas with small populations, with health plans receiving a pecuniary subsidy for material differences in fixed operational overheads and costs related to accessing services [34,43]. Rurality also nominally drove the development of Scotland’s “excess costs of supply” index. However, the index reflects the relative costs of supply across the country, irrespective of geographic classification, allowing for differential costs of providing services in either rural or urban areas [31].

A marked exception to the propensity to use rurality as the governing precept in these adjustments is England’s Market Forces Factor (MFF). Here the emphasis is on urbanity as a differential cost driver. In response to the higher costs associated with attracting and retaining the non-clinical workforce in certain parts of the country, the MFF is focused on restoring equilibrium in staffing costs between health plans in high living cost areas, particularly London and the South East of England, compared with lower cost areas [29].

#### Capitation adjustments

Bound by prospective budgets based on resident populations health plans are necessarily exposed to some level of risk where they are obligated to treat patients not included in their population base. Regional variation in the numbers of overseas visitors eligible for healthcare, but not included in the needs-based capitation, is of significant concern in New Zealand. In response the formula includes an ‘Overseas adjuster’ which acts to distribute a hypothecated sum between health plans according the actual costs historically incurred by each health plan due providing care to foreign visitors [43]. Balancing funding with anticipated population variation has also been a feature of England’s formula, where, testimony to the interdependence of health and social policy, the formula allowed for minor modifications to funding shares in areas where government policy was to increase housing supply via a ’growth area growth points’ adjustment. However, this adjustment has been removed from the formula in later funding rounds, following the discontinuation of the policy [29].

Patient flow between health plans can also have a substantial impact on a plan’s resources. At an operational level, several schemes have developed reciprocal arrangements between health plans to manage regional patient flows [44,45]. However, only NSW makes any adjustment for patient flow at a formula level, awarding a singular cost adjustment to the Southern Area Health Service (AHS) for the disproportionately high costs for interstate patient transfers charged by the neighbouring Australian Capital Territory AHS [26]. Deviating from the pattern amongst health systems with predominantly public provision, the overarching goal of preserving patient choice means Ontario circumvents these allocative complexities by distributing funds according to each health plan’s ‘market share’ of patients [35].

The presence of consumer choice in the Netherlands requires a different approach, as a result of health plans operating in a competitive environment. Despite conflicting with efficiency incentives, efforts to moderate negative risk selection, or ‘cream skimming’, by insurance funds has seen the Dutch formula allow retrospective adjustments to insurance companies for patients considerably in excess of expected costs [32].

### Unmet need

Although the primary rationale underlying needs-based formulae is the accurate prediction of health care expenditure, the ‘fair’ distribution of resources appeals to a concern for vertical equity–that those with the greatest need should receive the greatest share of resources. The utilisation driven structures common to all formulae, which inform the capitation components, act to promote equality of access based on demand. However, they risk reinforcing health disparities in groups that systematically under-utilise health services relative to their health needs. Since ‘unmet need’ is concealed by prevailing utilisation patterns, the implication is that formulae must engage in some form of normative comparison between sub-populations if equity of health outcomes is to be achieved. The approaches to addressing unmet need are described below.

#### Policy based measures

Only four of the formulas have explicitly attempted to address shortfalls between observed and expected utilisation by including some adjustment for unmet need (Table 5). Among these, two distinct approaches to identifying eligible populations have emerged. The first approach is to draw on a body of evidence pointing to poorer health outcomes in defined population groups. This has been the tactic adopted by New Zealand and NSW. In each case the significant and persistent health disparities experienced by indigenous and other deprived populations are recognised through ‘policy-weighted’ adjustments. NSW has achieved this by increasing the capitation weightings for Aboriginal and homeless populations to represent 2.5 times those of Other ethnicities as well as boosting allocations to the health plan exhibiting the highest need [26]. New Zealand has adopted a different approach, distributing a predetermined proportion of the global budget to health plans according to the proportion of Maori, Pacific and deprived populations resident in each district [34].

**Table 5:** Unmet need components included in the seven funding formulae

	***Policy based measure***	***Epidemiological based measure***
**England **[29]		Disability Free Life Expectancy (DFLE)
**Scotland **[31]		Rate of circulatory disease
**New South Wales **[26]	Indigenous and Homeless Population Weightings	
**Netherlands**		
**New Zealand **[34]	Maori, Pacific, NZ Index of Deprivation (NZDep2006) Quintiles 4&5 (40% most deprived areas)	
**Ontario**		
**Stockholm**		

Data sourced from:

[29] (Department of Health. 2011).

[31] (NHSScotland Resource Allocation Committee. 2007).

[26] (New South Wales Health. 2005).

[34] (Ministry of Health. 2004).

#### Epidemiological markers

The second approach to determining unmet need has been to use epidemiological markers. England and Scotland feature this approach, each carrying out extensive investigations to identify quantifiable, demonstrable and plausible indicators of unmet need. A requirement that any adjustment for unmet need be based on ‘clear definitions and robust methodology’ [31] underpins Scotland’s comparatively circumscribed adjustment, with allocations being adjusted for differential rates of circulatory disease. While still concerned that any adjustment for unmet need should be founded on robust epidemiological data, a measure of health expectancy has been seen as the most appropriate marker of unmet need in England [30]. By benchmarking each health plan’s Disability Free Life Expectancy score against a norm of seventy years, England captures both mortality and morbidity data to derive an ‘inequality weighted’ population. Despite extensive efforts, England has been unable to empirically establish the magnitude of any unmet need adjustment. As such the adjustment is determined as a proportion of the total budget, currently set at 10% [29].

## Discussion

This article has reviewed and compared population based funding formulae in seven health systems and sought to contextualise the formulae against common demographic and geographic features. In doing so, it contrasts with existing research into funding formulae [1,5-7] providing new insights into the relationships between population and health system factors and funding formulae parameters used in different jurisdictions.

Clearly, there are some similarities in how formulae are constructed, such as the factors that constitute the fundamental elements. For instance, basic demographic factors form a common heuristic solution to attributing expected expenditure. In common with previous studies [1,5], we found age, and in most cases, sex to be an elementary starting point. Similarly, factors reflecting socio-economic status are found across all formulae, although the level of refinement and the choice of area-level or individual-level data differs across all seven formulae. In contrast to these widely accepted elements, there is considerable variation around factors linked to unique challenges facing each health system. For example, ethnicity is included in health systems which exhibit manifest differences in health outcomes between different ethnic groups. This approach is also likely linked to explicit policy goals aimed at reducing ethnic disparities within these health systems [41,46]. In addition, there is considerable variation in the interpretation of geography in funding formulae, denoting the importance of the distribution of populations rather than overall population size. The interpretations of these factors suggest that the demographic heterogeneity of populations significantly influences the design of funding formulae.

In addition to demographic factors, a trend towards incorporating measures of disease into formulae denotes a proclivity for identifying more tangible indicators of need where reliable data are available. Within the formulae reviewed, two common formats are employed. In health systems where health plans are responsible for a geographically defined population epidemiological data acts as a proxy measure of population health. In contrast, in health systems where individual choice is a guiding tenet, a desire to minimise risk selection has led to individual-level clinical information being used to adjust allocations.

Recognition of cost pressures beyond those dictated by utilisation are also a common feature and are largely guided by population flux, dispersion and health system design.

A fundamental limitation confronting funding formulae is the difficulty delineating the relationships between demand, expenditure and genuine health need. The utilisation-driven structures common across all seven formulae risk reinforcing health disparities, yet given limited access to high quality data reflecting absolute need, adjustments for unmet need are captured in only a few formulae and are subject to divergent definitions.

## Conclusion

While an important reason for differences in interpretation of the above mentioned factors is the variation in observed relationships between factors in different jurisdictions, the context within which formulae are developed inevitably influences funding strategies. Although demonstrating significant parallels between approaches in the seven jurisdictions, our research suggests that, ultimately, the interpretation, design and implementation of the key structural elements within funding formulae are necessarily a function of wider political, socio-demographic and health system determinants, meaning there are also considerable differences among the seven approaches reviewed. This implies that formula construction, like most health policy issues, is partly a technical and partly contextual process [47]. As such, those searching for a one best method may draw some lessons from studying different health systems but find attempts to develop a formula will be dependent on data availability and the setting of goals and parameters by policy makers which, of course, is influenced by perceptions of public preferences.

With escalating pressure on health care resources, the requirement for a funding model to respond equitably to the needs of a dynamic and changing population means any formula will require ongoing refinement. Naturally, research into implications of alternative compositions will assist with this [3]. However, the comparative approach taken in this article also implies that further work is necessary in order to understand how well different population based funding formulae provide for the actual costs for health plans and, in turn, for health care needs.

Further research is needed to examine whether formulae in the seven systems compared in this article sufficiently represent and provide for the characteristics and health care needs accounted for. For example, the accuracy of Stockholm’s formula has recently been challenged as the descriptive variables’ predictive power was found to have diminished significantly since the formula was first devised. It was suggested that the addition of morbidity data was likely to yield a more accurate prediction of costs [37]. This research is consistent with a growing body of evidence which suggests that demographic data alone provide a poor explanation of variations in healthcare expenditure at both an individual level [48,49], as well as at a health plan level [50]. Such criticisms indicate that other funding formulae which rely heavily on demographic variables would benefit from further evaluation.

There is also a need for research into the all-important decisions that policy makers in different health systems make in terms of formula composition, including the strength of data and other information used for this. Correspondingly, greater understanding of the value of the different approaches to common issues is called for. For instance, despite adding depth to the overall portrait of need within geographic areas, epidemiological approaches to gauging differential need have been criticised for lacking sensitivity to regional variation in case mix and resource intensity [4]. Nevertheless, as epidemiological measures can be derived from routine or survey information they may provide a better reflection population health status than individual-based clinical measures which rely on interactions with health services and, as such, risk conflating utilisation with health need.

Finally, there is a need for research that probes whether any of the formulae used in different health systems performs sufficiently better than others at achieving similar policy goals, or whether the particular nuances that characterise individual country formulae are unlikely to improve or undermine comparative performance.

## Endnote

^a^“Health plan” is used in this article to describe arrangements where a funder or purchaser is responsible for a geographically based or enrolled population.

## Abbreviations

AHS: Area Health Service; MFF: Market Forces Factor; NSW: New South Wales (Australia); OECD: Organisation for Economic Co-operation and Development; SES: Socio-economic Status.

## Competing interests

The authors declare they have no competing interests.

## Authors’ contributions

All authors contributed equally to the conception and design of the study. All authors participated in drafting the manuscript. All authors read and approved the final manuscript.

## Pre-publication history

The pre-publication history for this paper can be accessed here:

http://www.biomedcentral.com/1472-6963/13/470/prepub
